# *Tremella fuciformis* Berk Alleviated Atherosclerosis Symptoms via Nuclear Factor-Kappa B-Mediated Inflammatory Response in ApoE^−/−^ Mice

**DOI:** 10.3390/nu17010160

**Published:** 2024-12-31

**Authors:** Yihao Dong, Qinchun Zhang, Rui Xie, Jundi Zhao, Zhihua Han, Yu Li, Han Yu, Yongfeng Zhang

**Affiliations:** 1 Engineering Research Center of Chinese Ministry of Education for Edible and Medicinal Fungi, Jilin Agricultural University, Changchun 130118, China; dongyihao@mails.jlau.edu.cn (Y.D.); zhangqinchun@mails.jlau.edu.cn (Q.Z.); xierui@mails.jlau.edu.cn (R.X.); zhaojundi@mails.jlau.edu.cn (J.Z.); hanzhihua@mails.jlau.edu.cn (Z.H.); liyv@jlau.edu.cn (Y.L.); 2 Tianjin Institute of Industrial Biotechnology, Chinese Academy of Sciences, Tianjin 300308, China; 3 National Center of Technology Innovation for Synthetic Biology, Tianjin 300308, China; 4 College of Agriculture, Jilin Agricultural University, Changchun 130118, China

**Keywords:** *Tremella fuciformis* Berk, atherosclerosis, NF-κB, gut microbiota, inflammatory response

## Abstract

Background: Atherosclerosis, a persistent inflammatory disease marked by the presence of atherosclerotic plaques or fibrous plaques, is a significant contributor to the onset of the development of cardiovascular disease. *Tremella fuciformis* Berk contains various active ingredients that have anti-inflammatory, antioxidant, and hypolipidemic properties. Nevertheless, the potential effects of *T. fuciformis* on atherosclerosis have not been systematically reported. Method: In this study, ApoE^−/−^ mice were employed as models of atherosclerosis caused by a high-fat diet (HFD) to investigate the effect of *T. fuciformis*. Gut microbiota and serum metabolism analysis were performed to elucidate the potential mechanism of *T. fuciformis* for its anti-atherosclerosis effects. Results: *T. fuciformis* significantly decreased the aortic root wall thickness and the area of lipid droplets, regulated lipid levels, and inhibited fat accumulation to improve aortic root lesions. Furthermore, *T. fuciformis* significantly altered serum metabolite (including diethyl phthalate and succinate) levels, regulated the abundance of microbiota, such as *Coriobacteriaceae_UCG-002* and *Alistipes*, and suppressed the inflammatory response to ameliorate atherosclerosis via the nuclear factor-kappa B (NF-κB)-mediated inflammatory response in HFD-induced ApoE^−/−^ mice. Conclusions: These results offer a theoretical basis and data to support *T. fuciformis* as a potential strategy for treating atherosclerosis.

## 1. Introduction

Atherosclerosis is a chronic inflammatory vascular disorder characterized by the formation of atherosclerotic plaques within the intima of arteries [1]. Atherosclerosis predominantly affects large and medium-sized arteries and can result in mortality and complications, including circulatory issues such as coronary artery diseases and cerebrovascular diseases [2]. Atherosclerosis significantly contributes to the onset and development of cardiovascular disease (CVD). In 2021, CVD accounted for 19.91 million global fatalities, and it is projected to cause 22.2 million deaths by 2030 [3,4]. The formation of atherosclerosis is correlated with a multitude of lifestyle factors, including hyperlipidemia, diabetes, smoking, staying up late, and obesity [3].

Atherosclerotic lesions are established through the chronic accumulation and subsequent deterioration of lipids, inflammatory cells, smooth muscle cells, and necrotic cell fragments beneath the endothelial intima [5]. The disorder of lipid metabolism is one of the primary detrimental factors affecting the development of atherosclerosis, resulting in the accumulation of cholesterol-rich smooth muscle cells and macrophages within the lining of blood vessels and the subsequent development of atherosclerotic plaques [6]. During the initial stages of atherosclerosis, the accumulation of oxidized low-density lipoprotein (LDL) in the aorta initiates the activation of arterial endothelial cells. This process encompasses inflammatory cells and factors, such as tumor necrosis factor-α (TNF-α) and interleukin-1β (IL-1β) [6,7]. Inflammatory cells and factors contribute to the development of atherosclerosis plaques [8]. The abnormal activation of nuclear factor-kappa B (NF-κB) leads to the up-regulation of TNF-α and IL-6, thereby facilitating the recruitment of monocytes in the aortic region and accelerating the progression of atherosclerotic plaques [9,10]. Furthermore, the presence and progression of arterial atherosclerosis are also influenced by the host’s gut microbiota [11]. The gut microbiota intervenes in a multitude of physiological processes within the host, encompassing the absorption and metabolism of lipids, inflammatory responses, and intestinal barrier formation [12,13]. The dysbiosis of gut microbiota could lead to alterations in the abundance of diverse products and metabolites, such as short-chain fatty acids (SCFAs) and lipopolysaccharide, thereby inducing an imbalance of the immune system, which gives rise to inflammatory responses in patients with atherosclerosis [14].

Statins are commonly used in the treatment of atherosclerosis [15]. However, the prolonged and repetitive utilization of these medications might give rise to adverse drug reactions, including liver damage and psychiatric adverse effects [16,17,18]. The diversity of active substances present in mushrooms renders the effective prevention and treatment of atherosclerosis theoretically feasible [19,20]. For instance, *Ganoderma lucidum* has manifested the capacity to alleviate atherosclerosis by its effects in alleviating endothelial dysfunction, reducing inflammation, and inducing the apoptosis of foam cells [21]. *Tremella fuciformis* Berk, also known as snow ear, belongs to the family Tremellaceae in the order Tremellales [22]. As a widely cultivated and popular mushroom species, *T. fuciformis* is extensively consumed as both food and a herbal ingredient, being recognized for its application as a traditional tonic in China [23,24]. *T. fuciformis* encompasses diverse active constituents, including polysaccharides, dietary fiber, and polyphenols [25]. The active components of *T. fuciformis* are responsible for its immunomodulatory activity [26], antioxidant properties [27], lipid-lowering activities [25], and anti-obesity [23]. However, the anti-atherosclerotic efficacy and underlying mechanisms of *T. fuciformis* have not been thoroughly investigated.

Apolipoprotein E (ApoE) regulates lipid transport and metabolism and cholesterol levels to maintain a balanced blood lipid profile [28]. ApoE^−/−^ mice are genetically engineered mouse models lacking the ApoE protein, which is essential for lipid metabolism, and they spontaneously develop hypercholesterolemia and atherosclerosis, making them a valuable tool for studying CVD and cholesterol-related disorders [29,30]. In this research, we established high-fat diet (HFD)-induced ApoE^−/−^ mice to investigate the potential mechanisms of *T. fuciformis* on atherosclerosis. Combined with gut microbiota and serum metabolomics analysis, *T. fuciformis* was found to regulate the structure of gut microbiota, alter metabolites, improve lipid metabolism, and inhibit aortic inflammation by regulating the NF-κB-mediated inflammatory response, thereby alleviating the symptoms of atherosclerosis. The results offer a theoretical basis and data to support *T. fuciformis* as a potential strategy for treating atherosclerosis.

## 2. Materials and Methods

### 2.1. Materials

*T. fuciformis* originated from Tongjiang (Bazhong, China) was dried, pulverized using a pulverizer (DF-Y-500C; Wenling Linda Machinery Co., Ltd., Taizhou, China), and subsequently sieved through an 80-mesh sieve to obtain *T. fuciformis* powder (TF).

### 2.2. Animals and Experiment Design

The experimental protocol was approved by the institutional ethics committee of Jilin Agricultural University (approval No. 20230908001) and conformed to the ARRIVE guidelines. Twenty-four male ApoE^−/−^ mice (8 weeks old) and eight male C57BL/6J mice (8 weeks old) were supplied by Hangzhou Ziyuan Laboratory Animal Science and Technology Co., Ltd. (Hangzhou, China) and maintained under specific-pathogen-free experimental conditions. Throughout the experimental period, mice had unrestricted access to adequate food and water.

**Figure 1:**
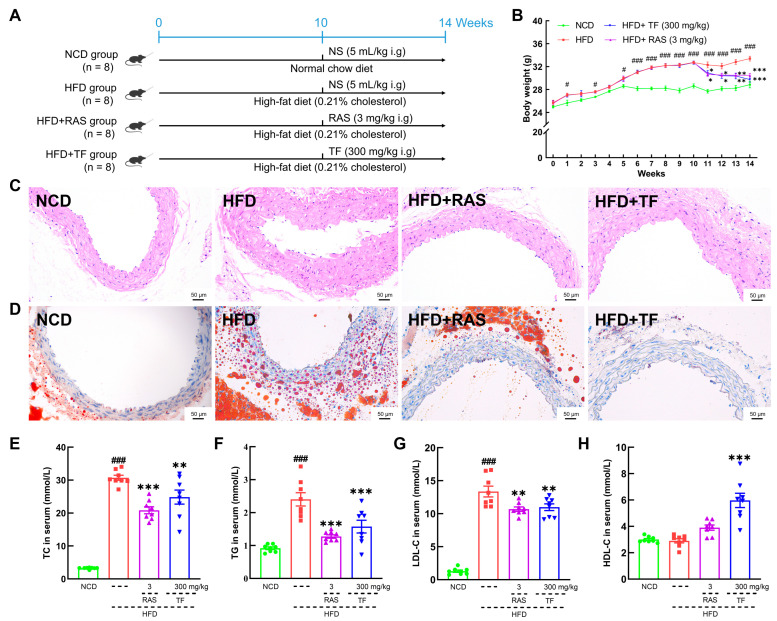
*T. fuciformis* ameliorated atherosclerosis symptoms in HFD-induced ApoE^−/−^ mice. (**A**) The HFD-induced mice establishment and treatment processes. (**B**) *T. fuciformis* inhibited body weight gain in HFD-induced ApoE^−/−^ mice (n = 8). Histopathological analysis of the aortic root by (**C**) H&E stain and (**D**) oil red O stain (200×; scale bars = 50 µm) (n = 3). *T. fuciformis* reduced serum levels of (**E**) TC, (**F**) TG, and (**G**) LDL-C and increased the level of (**H**) HDL-C (n = 8). All data are presented as mean ± S.E.M. ^#^ *p* < 0.05, ^###^ *p* < 0.001 vs. NCD mice; * *p* < 0.05, ** *p* < 0.01, *** *p* < 0.001 vs. HFD-induced ApoE^−/−^ mice.

The establishment and treatment processes of the HFD-induced ApoE^−/−^ mice model are presented in Figure 1A. Following acclimatization, ApoE^−/−^ mice were provided with HFD, while C57BL/6J mice were fed normal chow diet (NCD) for 14 weeks. (The details of the ingredients of HFD and NCD are presented in Table S1). From the beginning of the 11th week, the ApoE^−/−^ mice were allocated randomly into three groups: the HFD group (n = 8, normal saline (NS) 5 mL/kg), the HFD+RAS group (n = 8, Rosuvastatin (RAS) 3 mg/kg) and the HFD+TF group (n = 8, TF 300 mg/kg), and the C57BL/6J mice served as the NCD group (n = 8, NS 5 mL/kg). NS, RAS, and TF were orally administered daily for 4 weeks. At the end of the experiment, mice were weighed and euthanized after fasting for 12 h. Subsequently, the aortic root, blood, cecum contents, and four types of adipose tissues (eWAT, iWAT, pWAT, and BAT) were collected, along with other organs (liver, heart, kidney, spleen, and thymus), for pathological and biochemical analyses.

### 2.3. Organ and Adipose Tissue Indices Analysis

The collected adipose tissue and major organs were weighed, and their indexes were calculated using the following formulas: organ or adipose tissue index = organ or adipose tissue weight (g)/body weight (g) × 100%.

### 2.4. Histological Analysis

The aortic root, adipose tissues, and organs mentioned above were fixed in 4% paraformaldehyde for more than 24 h. Subsequently, these partial aortic roots, adipose tissue, and organs were processed (dehydrated, embedded, and sectioned) to obtain 4 μm thick sections in paraffin. Then, the tissue sections were subjected to staining using H&E. Additionally, the remaining aortic root tissues were processed (dehydrated in a sucrose solution, embedded with optimal cutting temperature compound, frozen quickly, and sectioned) to obtain 8 μm thick cryosections, and then stained with oil red O and hematoxylin. All slides were observed under a microscope equipped with an imaging system (NIKON ECLIPSE CI, Nikon, Tokyo, Japan) [31].

### 2.5. Immunofluorescence Staining

After preconditioning (dehydration, permeabilization, blocking, antigen retrieval), the aortic root sections were incubated with the anti-NLRP3 antibody overnight. Subsequently, they were incubated with a secondary fluorescence-labeled antibody for 50 min at 25 °C in the dark, followed by staining with DAPI to label the nuclei. The immunofluorescence results were observed using a microscope equipped with an imaging system (NIKON ECLIPSE CI, Nikon, Tokyo, Japan) and then quantified and analyzed using ImageJ software (ImageJ 1.54g, NIH, Bethesda, MD, USA) [32]. The antibody information is displayed in Table S2.

### 2.6. Serum Lipid Detection

The collected blood samples were separated at 3500 rpm for 15 min twice to obtain serum. The contents of serum lipids were detected using commercially available assay kits (details of the kits are shown in Table S3), as previously described [33].

### 2.7. Gut Microbiota Analysis

The 16s rRNA was amplified using primer 341F-806R (details are shown in Table S4) from DNA extracted from the cecal contents of NCD, HFD, and HFD+TF mice. Sequencing was performed using the Illumina NovaSeq6000 platform (Illumina, San Diego, CA, USA). The obtained sequences were quality filtered with fastp software (v0.23.0, HaploX, Shenzhen, China) and merged with Fast Length Adjustment of Short reads (FLASH) software (v1.2.11). The sequences were all grouped into variants based on amplicon sequences (ASVs). Using the flattened ASVs, a range of diversity indices were analyzed, and the sequencing depth was assessed. Additionally, the community composition was statistically evaluated using taxonomic data at different levels of taxonomy [34].

### 2.8. Non-Targeted Metabolomics Analysis

Serum samples collected from the NCD, HFD, and HFD+TF mice were analyzed after preprocessing, as previously described [35]. The raw data were preprocessed (peak picking, peak grouping, normalized) using XCMS software (v3.1.0) to obtain the data. Subsequently, the isotope data were removed. Data were processed using the 80% rule and relative standard deviation >30% in quality control samples. VIP value > 1 and *p* < 0.05 was considered statistically significant.

### 2.9. Western Blot

The aortic root tissue was lysed with a mixture of 10% radioimmunoprecipitation assay buffer and 1% protease and phosphatase inhibitors at 4 °C. Subsequently, the lysate was centrifuged (12,000 rpm, 5 min) to obtain the supernatant. The protein concentration in the supernatants was quantified using the Pierce™ BCA Protein Assay Kit (Thermo Fisher Scientific, Waltham, MA, USA). The separation of proteins was achieved using 10% sodium dodecyl sulfate–polyacrylamide gel electrophoresis, followed by subsequent transfer onto polyvinylidene fluoride membranes. After blocking, the membranes were incubated with primary antibodies (including GPR91, hypoxia-induced factor-1α (HIF-1α), P-NF-κB, T-NF-κB, P-IKKα+β, T-IKKα+β, P-IκBα, T-IκBα, NLRP3, IL-6, IL-1β, TNF-α, IL-10, and GAPDH) at 4 °C overnight, and then incubated with a secondary antibody for 4 h (details of antibodies are displayed in Table S2). Finally, the bands were visualized with the chemiluminescence imaging system and then quantified and analyzed using the ImageJ software (ImageJ 1.54g, NIH, Bethesda, MD, USA) [33].

### 2.10. Statistical Analysis

All data are presented as mean ± Standard Error of the Mean (S.E.M.). SPSS Statistics 26 software (IBM, Armonk, NY, USA) was used for statistical analyses, and GraphPad Prism 9 software (GraphPad Software Inc., San Diego, CA, USA) was used to generate graphs. One-way analysis of variance (One-Way ANOVA) followed by Dunnett’s *t*-test (2-sided) was performed. Statistical significance was *p* < 0.05.

## 3. Results

### 3.1. T. fuciformis Alleviated Atherosclerosis Progression in HFD-Induced ApoE^−/−^ Mice

**Figure 2:**
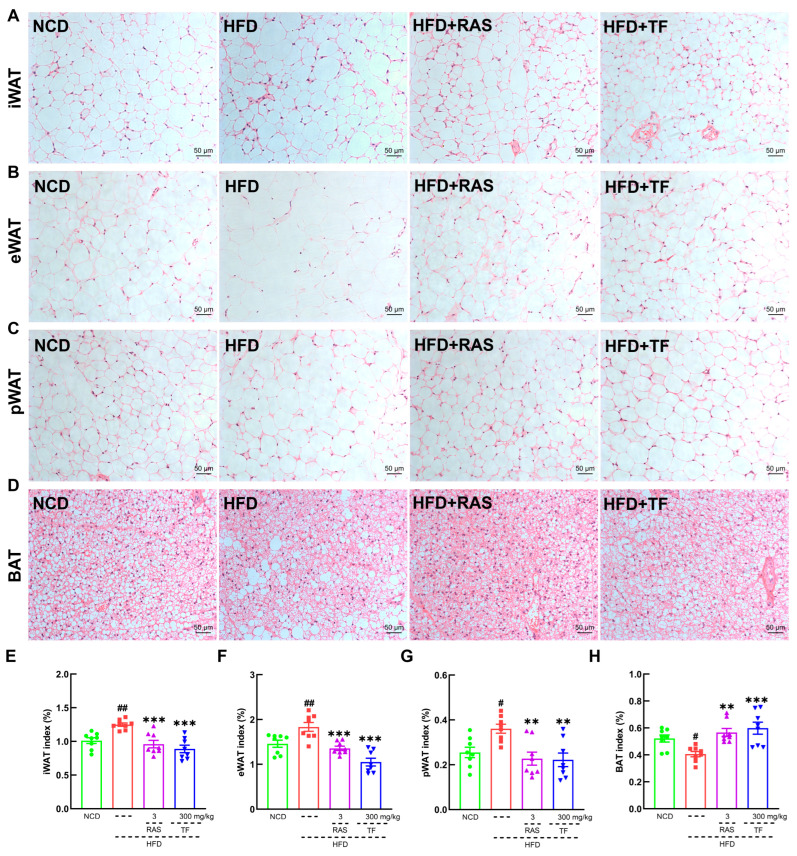
*T. fuciformis* attenuated fat accumulations in HFD-induced ApoE^−/−^ mice. Histopathological analysis of (**A**) iWAT, (**B**) eWAT, (**C**) pWAT, and (**D**) BAT by H&E staining: (200×; scale bars = 50 µm) (n = 3). *T. fuciformis* decreased the indexes of (**E**) iWAT, (**F**) eWAT, and (**G**) pWAT and increased the index of (**H**) BAT (n = 8). All data are expressed as mean ± S.E.M. ^#^ *p* < 0.05, ^##^ *p* < 0.01 vs. NCD mice. ** *p* < 0.01, *** *p* < 0.001 vs. HFD-induced ApoE^−/−^ mice.

To investigate the efficacy of *T. fuciformis* on atherosclerosis development, ApoE^−/−^ mice fed an HFD were orally administered with TF daily for a period of 4 weeks (Figure 1A). Compared with the NCD-fed mice, long-term HFD consumption in ApoE^−/−^ mice resulted in enhanced weight gain (*p* < 0.001; Figure 1B), promoted the development of aortic root lesions, and increased aortic root wall thickness (Figure 1C), as well as enlarged the area of lipid droplets on the vascular wall at the aortic root (Figure 1D). These effects were effectively reversed by four weeks of RAS and TF treatment. Additionally, lipid-driven chronic inflammation has a negative impact on atherosclerosis, and alleviation of hyperlipidemia has become the mainstay of treatment for atherosclerosis [36]. In comparison to NCD-fed mice, the serum levels of TC (*p* < 0.001; Figure 1E), TG (*p* < 0.001; Figure 1F), and LDL-C (*p* < 0.001; Figure 1G) were significantly increased in HFD-induced ApoE^−/−^ mice. Treatment with TF and RAS inhibited the serum levels of TC (*p* < 0.01; Figure 1E), TG (*p* < 0.001; Figure 1F), and LDL-C (*p* < 0.01; Figure 1G) in HFD-induced ApoE^−/−^ mice. Interestingly, HFD intake and RAS treatment showed no significant impact on altering the serum levels of HDL-C; however, TF treatment increased the serum HDL-C levels (*p* < 0.001; Figure 1H) in HFD-induced ApoE^−/−^ mice. Moreover, TF and RAS treatment inhibited adipocyte hypertrophy and vacuolization in iWAT (Figure 2A), eWAT (Figure 2B), and BAT (Figure 2D), but TF did not inhibit adipocyte hypertrophy and vacuolization in pWAT (Figure 2C) in HFD-induced ApoE^−/−^ mice. The indexes of iWAT (*p* < 0.01; Figure 2E), eWAT (*p* < 0.01; Figure 2F), and pWAT (*p* < 0.05; Figure 2G) were enhanced, while the BAT index (*p* < 0.05; Figure 2H) was strongly decreased in HFD-induced ApoE^−/−^ mice compared to NCD-fed mice, which were significantly reversed by TF and RAS treatment. Overall, these results suggested that *T. fuciformis* alleviated atherosclerosis progression.

In addition, further exploration was conducted into the pathological effects of TF on major organs, as well as its impact on organ indexes. The histopathological results revealed that TF suppressed lipid vacuole accumulation in the liver (Figure S1A), improved the disorder of white and red medulla in the spleen (Figure S1B), and had no effect on kidney (Figure S1C), heart (Figure S1D), and thymus (Figure S1E) in HFD-induced ApoE^−/−^ mice. Furthermore, TF treatment reduced the indexes of the liver (*p* < 0.001; Figure S2A), heart (*p* < 0.01; Figure S2B), and kidney (*p* < 0.001; Figure S2C) without affecting the spleen (Figure S2D) and thymus (Figure S2E) in HFD-induced ApoE^−/−^ mice.

### 3.2. T. fuciformis Regulated Gut Microbes in HFD-Induced ApoE^−/−^ Mice

**Figure 3:**
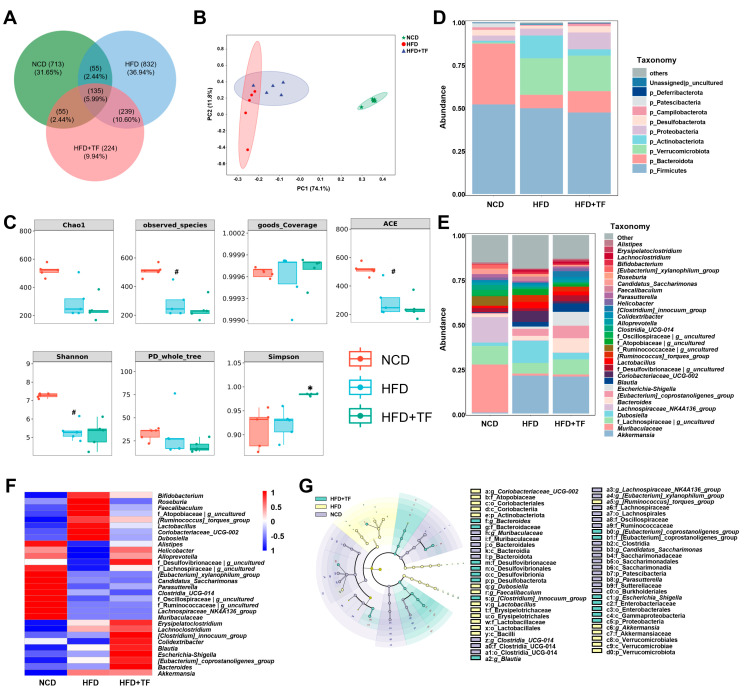
*T. fuciformis* altered the composition of the gut microbiota in HFD-induced ApoE^−/−^ mice. (**A**) Venn diagram based on the ASV distribution. (**B**) β-diversity. (**C**) α-diversity. (**D**) Histogram of different species at the phylum level at top 10. (**E**) Histogram of different species at the genus level at top 30. (**F**) Heatmap of differential species at the genus level at the top 30. (**G**) Taxonomic lineage map based on LEfSe analysis. Nodes of different colors represent microbial taxa that play important roles in the corresponding groups (n = 5). ^#^ *p* < 0.05 vs. NCD mice. * *p* < 0.05 vs. HFD-induced ApoE^−/−^ mice.

The gut microbiota and the substances it produces are recognized as having a substantial impact on the progression of atherosclerosis [14]. Thus, gut microbial diversity was assessed to explore the shift in gut microbes of HFD-induced ApoE^−/−^ mice following *T. fuciformis* treatment. A total of 2253 ASVs were identified, with the distribution as follows: 958 within the NCD group, 1261 within the HFD group, and 653 within the HFD+TF group, of which the number of specific ASVs was 713, 832, and 224, respectively (Figure 3A). The β-diversity results suggested clear clustering of microbiota composition between HFD-induced ApoE^−/−^ mice and NCD-fed C57BL/6J mice; however, the microbial composition of mice treated with *T. fuciformis* showed limited differentiation from that of the HFD-induced ApoE^−/−^ mice (Figure 3B). The α-diversity indexes indicated that prolonged-term HFD consumption definitely reduced gut microbiota diversity in ApoE^−/−^ mice, as evidenced by decreased observed_species, ACE, and Shannon indices, whereas *T. fuciformis* could apparently elevate the Simpson indices in HFD-induced ApoE^−/−^ mice (Figure 3C). At the phylum level, *T. fuciformis* treatment increased the relative abundance of p_Bacteroidota and decreased the relative abundance of p_Firmicutes in HFD-induced ApoE^−/−^ mice (Figure 3D). At the genus level in the top 30, compared with the HFD group, *T. fuciformis* treatment reduced the relative abundance of eight types, such as *Coriobacteriaceae_UCG-002*, and increased the relative abundance of five types, including *Alistipes* (Figure 3E,F). LEfSe analysis showed that the significant genus enrichments in the HFD-induced ApoE^−/−^ mice treated with *T. fuciformis* were *Bacteroides*, *[Clostridium]_innocuum_group*, *Blautia*, *Escherichia_Shigella*, *[Eubacterium]_coprostanoligenes_group* (Figure 3G).

### 3.3. T. fuciformis Altered Serum Metabolite Levels in HFD-Induced ApoE^−/−^ Mice

**Figure 4:**
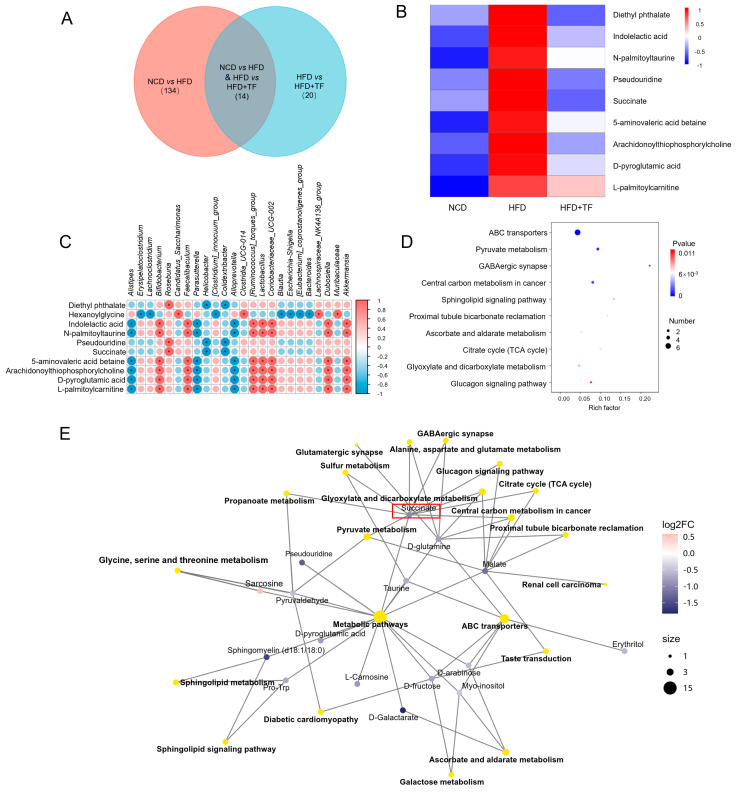
*T. fuciformis* regulated the serum metabolites in HFD-induced ApoE^−/−^ mice. (**A**) Venn diagram. (**B**) Heatmap of serum differential metabolites in each group. (**C**) Spearman correlation analysis of differential gut microbiota and significantly differential metabolites. Red is a positive correlation, and blue is a negative correlation. (* *p* < 0.05). (**D**) KEGG enrichment pathway analysis of different metabolites. (**E**) Network diagrams of the KEGG pathways and metabolites (n = 5).

To further elucidate the potential mechanisms of *T. fuciformis* in alleviating atherosclerosis, the serum content from NCD, HFD, and HFD+TF mice was analyzed by non-targeted metabolomics. A total of 148 differential metabolites were found between NCD and HFD groups, and 34 differential metabolites were found between HFD and HFD+TF groups, of which the number of specific differential metabolites was 134 and 20, respectively (Figure 4A). Compared with the NCD group, HFD up-regulated the levels of diethyl phthalate, indolelactic acid, N-palmitoyltaurine, pseudouridine, succinate, 5-aminovaleric acid betaine, arachidonoylthiophosphorylcholine, D-pyroglutamic acid, and L-palmitoylcarnitine, and the treatment of *T. fuciformis* reversed these changes in up-regulation (Figure 4B). Spearman’s correlation analysis of the significantly different microbiota and metabolites showed an obvious positive correlation between the level of succinate and diethyl phthalate and the abundance of *Roseburia* (*p* < 0.05, Figure 4C). The Kyoto Encyclopedia of Genes and Genomes (KEGG) pathway enrichment analyses of the HFD and HFD+TF groups showed that different metabolites were mainly involved in the citric acid cycle (TCA cycle) (Figure 4D). Notably, the KEGG pathway network diagram visually demonstrated a connection between the differential metabolite succinate and the TCA cycle (Figure 4E). The results suggest that the potential mechanism of *T. fuciformis* in alleviating atherosclerosis may be related to succinate-mediated inflammation factors.

### 3.4. T. fuciformis Alleviated Aortic Inflammation via NF-κB Signaling Pathway in HFD-Induced ApoE^−/−^ Mice

**Figure 5:**
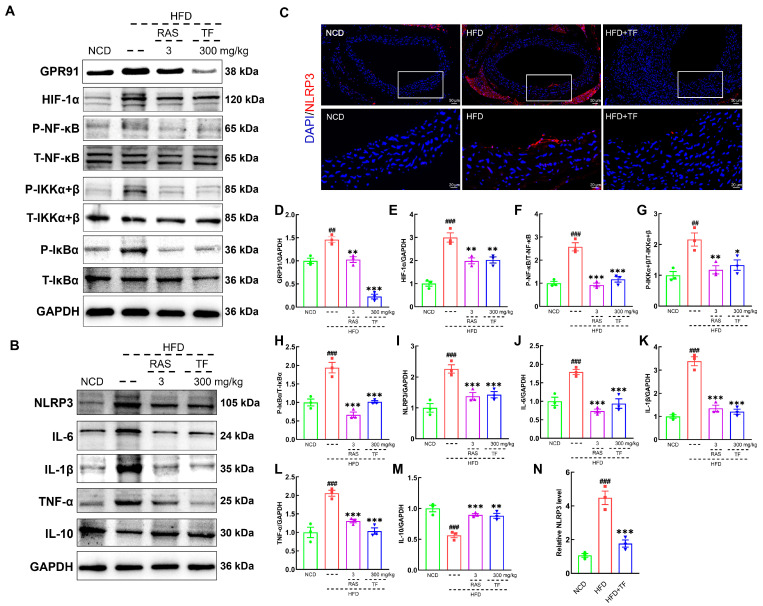
*T. fuciformis*-regulated NF-κB signaling pathway in HFD-induced ApoE^−/−^ mice. (**A**) Western blot analysis of GPR91, HIF-1α, P-NF-κB, T-NF-κB, P-IKKα+β, T-IKKα+β, P-IκBα, and T-IκBα expression in the aorta root (n = 3). (**B**) Western blot analysis of NLRP3, IL-6, IL-1β, TNF-α, and IL-10 expression in the aorta root (n = 3). (**C**) Immunofluorescence staining of NLRP3 (red) and DAPI (blue) in the aorta root (200×; scale bar: 50 μm) (n = 3). (**D**–**M**) Semi-quantitative analysis of protein expression in A and B was normalized to GAPDH and manifested as the fold of the NCD group. (**N**) Semi-quantification of NLRP3. ^##^ *p* < 0.01 and ^###^ *p* < 0.001 vs. NCD mice, * *p* < 0.05, ** *p* < 0.01 and *** *p* < 0.001 vs. HFD-induced ApoE^−/−^ mice.

The activation of NF-κB can recruit inflammatory cell infiltration and promote the production of inflammatory mediators, thereby exacerbating the inflammatory response in vascular walls and plaque formation in atherosclerotic diseases [37]. *T. fuciformis* and RAS treatment significantly suppressed the expressions of GPR91 (*p* < 0.001; Figure 5A,D), HIF-1α (*p* < 0.01; Figure 5A,E), P-NF-κB (*p* < 0.001; Figure 5A,F), P-IKKα+β (*p* < 0.05; Figure 5A,G), and P-IκBα (*p* < 0.001; Figure 5A,H) in the aortic root of HFD-induced ApoE^−/−^ mice. In addition, NF-κB activation and nuclear translocation can promote NLRP3 expression [38]. *T. fuciformis* and RAS treatment effectively decreased the contents of NLRP3 (*p* < 0.001; Figure 5B,I), IL-6 (*p* < 0.001; Figure 5B,J), IL-1β (*p* < 0.001; Figure 5B,K), and TNF-α (*p* < 0.001; Figure 5B,L) and increased the IL-10 levels (*p* < 0.01; Figure 5B,M) in the aortic root of HFD-induced ApoE^−/−^ mice. The suppressive effect of *T. fuciformis* on the expression of NLRP3 in the aortic root was also validated by the IF analysis (*p* < 0.001; Figure 5C,N). The findings suggested that *T. fuciformis* treatment suppressed the NF-κB-mediated inflammatory response to regulated inflammation in HFD-induced ApoE^−/−^ mice.

## 4. Discussion

As a chronic metabolic disorder, atherosclerosis is influenced by disturbances in lipid metabolism [39]. In dyslipidemia, elevated levels of TG, total TC, and LDL in the bloodstream are significant detrimental factors that contribute to the formation of atherosclerotic plaques [40,41,42]. The accumulation of oxidized LDL within the vascular endothelium will expedite the formation of macrophage foam cells in HFD-induced ApoE^−/−^ mice [43]. Furthermore, heightened levels of TC lead to the accumulation of cholesterol in macrophages and smooth muscle cells, thereby promoting plaque formation [44]. Elevated levels of TG lead to the deposition of TC within the endothelial cells that line the arterial wall, subsequently inducing endothelial inflammation [45]. In this study, *T. fuciformis* significantly decreased the aortic root wall thickness and the area of lipid droplets and down-regulated serum lipid (TG, TC, and LDL-C) levels in HFD-induced ApoE^−/−^ mice. Simultaneously, *T. fuciformis* mitigated adipocyte hypertrophy and vacuolation in both iWAT and eWAT, as well as BAT, thereby suppressing fat accumulation induced by an HFD in ApoE^−/−^ mice. Thus, these results suggest that *T. fuciformis* regulated lipid metabolism factors to suppress the atherosclerosis process in HFD-induced ApoE^−/−^ mice.

As an essential “organ” of the host organism, the gut microbiota and its metabolites affect the development of atherosclerotic plaques by regulating lipid levels and inflammatory reactions [14]. Bacteroidota directly reduce the adhesion of detrimental bacteria in the intestinal tract and prevent the imbalance of the gut microbiota [46]. In contrast, Firmicutes facilitate the absorption of energy from food, thereby contributing to the accumulation of lipids and the development of atherosclerosis in the host [47]. Our results showed that *T. fuciformis* enhanced the abundance of Bacteroidota and decreased the abundance of Firmicutes. At the genus level, *T. fuciformis* up-regulated the abundance of *Alistipes* and down-regulated the abundance of *Coriobacteriaceae_UCG-002* in the cecum contents of HFD-induced ApoE^−/−^ mice. *Alistipes* produces propionate [48], which decreases the entry of acetate into liver cells, thereby reducing cholesterol synthesis and ultimately indirectly inhibiting the formation of atherosclerotic plaques [49]. *Coriobacteriaceae_UCG-002* promotes the development of atherosclerotic plaques through modulating hepatic metabolism and intestinal cholesterol levels, which is correlated with the regulation of proprotein convertase subtilisin/kexin type 9 expression in the liver and ABCA1 expression in the gastrointestinal tract by *Coriobacteriaceae_UCG-002* [50,51]. *Bacteroides* strengthens the integrity of the intestinal barrier and decreases the translocation of LPS from the gut microbiota to the bloodstream, thereby alleviating LPS-induced endotoxemia and intensifying the NF-κB inflammatory response within atherosclerotic plaques [52]. Notably, LEfSe analysis revealed that *T. fuciformis* significantly enriched the content of *Bacteroides* in HFD-induced ApoE^−/−^ mice. Thus, *T. fuciformis* might have the ability to regulate gut microbiota disorders, thereby regulating inflammation and lipid metabolism factors in HFD-induced ApoE^−/−^ mice.

The host’s metabolism is affected by the dysbiosis of the gut microbiota, which results in alterations in the composition and concentrations of metabolic compounds within the host [12]. Furthermore, the modifications in specific metabolites elicited by the gut microbiota regulate the advancement of atherosclerotic plaque and instigate detrimental inflammation in the aortic endothelium [53,54]. Prolonged exposure to diethyl phthalate elicits oxidative stress and inflammatory responses in adipose tissue, thereby resulting in disturbances in lipid metabolism and indirectly promoting the development of atherosclerotic plaques [55,56]. Succinate acts as a vital intermediary in the tricarboxylic acid (TCA) cycle, exerting a crucial impact on cellular energy metabolism and biosynthesis [57]. Disruption of the TCA cycle homeostasis results in an excessive efflux of succinate from the mitochondrial matrix, thereby disturbing the balance between intracellular and extracellular environments [58]. Extracellular succinic acid stimulates succinate receptor 1 (GPR91, also known as SUCNR1) in endothelial cells and macrophages to stabilize HIF-1α, thereby activating NLRP3 and increasing the expression of IL-1β [58]. In our study, *T. fuciformis* reduced the concentrations of diethyl phthalate and succinate in HFD-induced ApoE^−/−^ mice. Meanwhile, *T. fuciformis* exerted a significant influence on TCA circulation. Therefore, *T. fuciformis* might regulate serum metabolism factors to influence the inflammatory response in HFD-induced ApoE^−/−^ mice.

Chronic low-grade inflammation, a crucial driver of atherosclerosis, is characterized by multiple signaling pathways regulated by immune and inflammatory mediators [59]. Throughout the progression of atherosclerosis, TNF-α promotes the translocation of LDL to the subendothelial layer of blood vessel walls, leading to its accumulation and subsequent oxidation to oxidized LDL [60]. IL-10 mainly serves to inhibit the activation of macrophages and reduce the secretion of pro-inflammatory cytokines in foam cells derived from lipid-filled and activated macrophages [61]. IL-6 possesses the ability to up-regulate the expression of CD36 in macrophages and augment the uptake of oxidized LDL by macrophages, thereby facilitating the generation of macrophage foam cells [62]. The production and release of intracellular IL-6 are facilitated by inflammatory stimuli, a process regulated by NF-κB and IKKα+β in macrophages [63]. Inflammatory stimuli trigger the phosphorylation of IκBα via the activation of IKKα+β, thereby promoting the release and nuclear translocation of NF-κB [64]. Consequently, IKKα+β governs the expression of a multitude of inflammation-related genes within cells and modulates inflammatory responses by regulating the NF-κB signaling pathway [65]. Activated NF-κB is translocated to the nucleus, within which it promotes the expression of NLRP3 and inflammatory cytokine genes [66]. Subsequently, NLRP3 can be activated by various inflammatory factors in response to cellular stress [67]. This activation of the molecules initiates the assembly of inflammasomes and subsequently leads to the activation of Caspase-1, which, in turn, triggers the conversion of IL-1β precursor to IL-1β [68]. In the initial stages of atherosclerosis, IL-1β triggers inflammation in the aortic endothelium and promotes the recruitment of inflammatory cells into the blood vessels, leading to their invasion of the intima [69]. In our study, *T. fuciformis* was able to regulate the NF-κB signaling pathway, thereby regulating the inflammation factors in HFD-induced ApoE^−/−^ mice.

However, the experiment retained certain limitations. Firstly, *T. fuciformis* has multiple active components, such as polysaccharides, proteins, and polyphenols; therefore, it is necessary to further explore which active component plays the primary role in HFD-induced ApoE^−/−^ mice. Secondly, although it has been preliminarily confirmed that *T. fuciformis* regulates the NF-κB signaling pathway and the gut microbiota in HFD-fed ApoE^−/−^ mice, the regulatory relationship between NF-κB and the gut microbiota still needs further investigation.

## 5. Conclusions

Our research demonstrated that *T. fuciformis* could modulate serum lipid levels, alter the composition of gut microbiota, and inhibit the inflammatory response to ameliorate atherosclerotic processes via the suppression of the NF-κB signaling pathway in HFD-induced ApoE^−/−^ mice. These results provide a theoretical foundation and data for considering *T. fuciformis* as a potential strategy for treating atherosclerosis.

## Data Availability

The original contributions presented in this study are included in the article/Supplementary Materials. Further inquiries can be directed to the corresponding author.

## Supplementary Materials

The following supporting information can be downloaded at: https://www.mdpi.com/article/10.3390/nu17010160/s1, Figure S1. H&E staining of liver (A), spleen (B), kidney (C), heart (D), and thymus (E) (100×; scale bar: 100 μm); Figure S2. The effect of *T. fuciformis* on organ indexes in HFD-induced ApoE^−/−^ mice. The indexes of the liver (A), heart (B), kidney (C), spleen (D), and thymus (E). All data are expressed as mean ± S.E.M. ^##^ *p* < 0.01, ^###^ *p* < 0.001 vs. NCD mice. * *p* < 0.05, ** *p* < 0.01, *** *p* < 0.001 vs. HFD-induced ApoE^−/−^ mice; Table S1. Details of HFD and NCD ingredients; Table S2. Details of antibodies used in Western blot and immunofluorescence; Table S3. Details of commercially available test kits; Table S4. Details of primer 341F-806R; Table S5. Relative abundance (log10) of top 10 phyla among NCD, HFD, and HFD+TF groups. Data are presented as the mean; Table S6. Relative abundance (log10) of the top 30 genera among NCD, HFD, and HFD+TF groups. Data are presented as the mean; Table S7. The differential metabolites of serum among NCD, HFD, and HFD+TF groups. Data are presented as the mean. Differences were considered statistically significant at *p* < 0.05 and VIP > 1.
