# Supplementary material for: Tremella fuciformis Berk Alleviated Atherosclerosis Symptoms via Nuclear Factor-Kappa B-Mediated Inflammatory Response in ApoE−/− Mice
Source: Nutrients. 2024 Dec 31;17(1):160. doi: 10.3390/nu17010160 (PMC11722796; doi:10.3390/nu17010160)
Supplement: Supplementary file 1 [file nutrients-17-00160-s001.zip › nutrients-3385198-Supplementary.pdf]

Tremella fuciformis Berk alleviated atherosclerosis symptoms via NF-κB-mediated inflammatory response in ApoE$^{-/-}$ mice.

Yihao Dong [1], Qinchun Zhang [1], Rui Xie [1], Jundi Zhao [1], Zhihua Han [1], Yu Li [1,2,3], Han Yu [4,*], and Yongfeng Zhang [1,*]

[1]*Engineering Research Center of Chinese Ministry of Education for Edible and Medicinal Fungi, Jilin Agricultural University, Changchun 130118, China*
[2]*Tianjin Institute of Industrial Biotechnology, Chinese Academy of Sciences, Tianjin 300308, China*
[3]*National Center of Technology Innovation for Synthetic Biology, Tianjin 300308, China*
[4]*College of Agriculture, Jilin Agricultural University, Changchun 130118, China*
[*]Correspondence: yuhan@jlau.edu.cn (H.Y.); zhangyongfeng@jlau.edu.cn (Y.F.)

Han Yu, Professor, Ph.D
College of Agriculture, Jilin Agricultural University,
Changchun, Jilin, P.R.China.
Email: yuhan@jlau.edu.cn

Yongfeng Zhang, Ph.D
Engineering Research Center of Chinese Ministry of Education for Edible and Medicinal Fungi, Jilin Agricultural University,
Changchun, Jilin, P.R.China.
Email: zhangyongfeng@jlau.edu.cn

Supplementary Figures

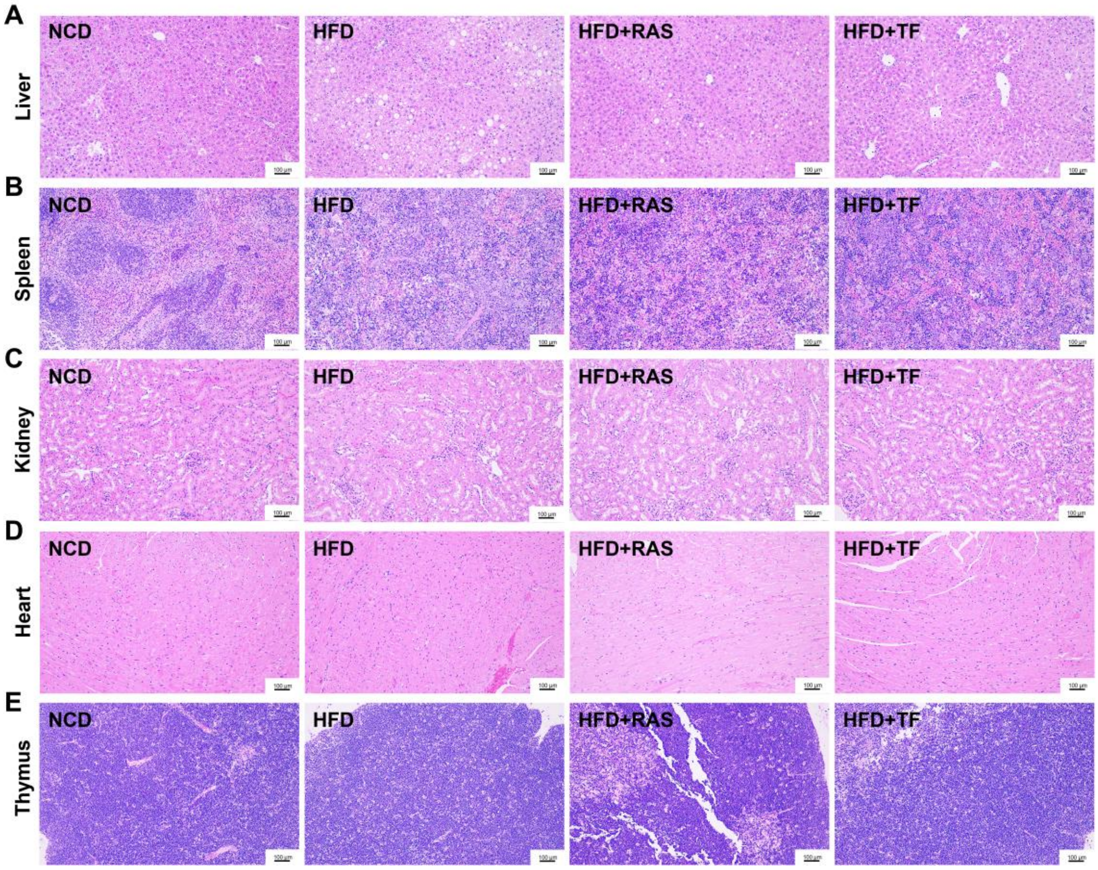

Figure S1. H&E staining of liver (A), spleen (B), kidney (C), heart (D), and thymus (E) (100×; scale bar: 100 μm).

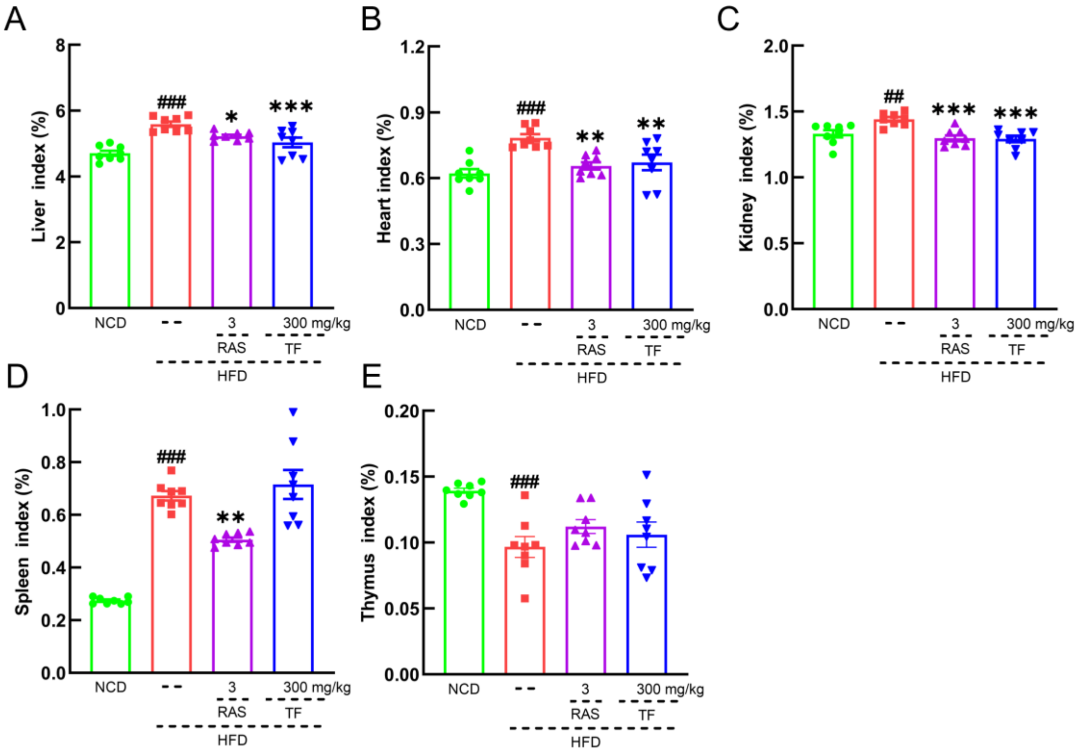

Figure S2. The effect of *T. fuciformis* on organ indexes in HFD-induced ApoE$^{-/-}$ mice. The indexes of the liver (A), heart (B), kidney (C), spleen (D), and thymus (E). All data are expressed as mean ± S.E.M. $^{\#\#}p < 0.01$, $^{\#\#\#}p < 0.001$ *vs* NCD mice. $^{*}p < 0.05$, $^{**}p < 0.01$, $^{***}p < 0.001$ *vs* HFD-induced ApoE$^{-/-}$ mice.

Supplementary Tables

Table S1. Details of HFD and NCD ingredients.

	NCD	HFD
Fat (kcal%)	10	40
Protein (kcal%)	20	17
Carbohydrate (kcal%)	60	43
cholesterol (%)	--	0.21

Table S2. Details of antibodies used in western blot and immunofluorescence.

Antibody	Molecular weight	Catalog number	Dilution for Western Blot	Dilution for Immunofluorescence	Company	Area
NLRP3	105 kDa	BA3677	--	1:200	Boster	Wuhan, China
GPR91	38 kDa	AF5316	1:1000	--	Affinity	Cincinnati, OH, USA
HIF-1α	120 kDa	A16873	1:1000	--	ABclonal	Wuhan, China
P-NF-κB	65 kDa	AF3387	1:1000	--	Affinity	Cincinnati, OH, USA
T-NF-κB	65 kDa	AF5006	1:1000	--	Affinity	Cincinnati, OH, USA
P-IKKα+β	85 kDa	2697S	1：1000	--	Cell Signaling	Beverly, MA, USA
T-IKKα+β	85 kDa	AF6014	1：1000	--	Affinity	Cincinnati, OH, USA
P-IKBα	36 kDa	ab133462	1:25000	--	Abcam	Cambridge, MA, USA
T-IKBα	36 kDa	AF7776	1:1000	--	Affinity	Cincinnati, OH, USA
GAPDH	36 kDa	E-AB-40337	1:1000	--	Elabscience	Wuhan, China
NLRP3	105 kDa	A12694	1:2000	--	ABclonal	Wuhan, China
IL-6	24 kDa	DF6087	1:1000	--	Affinity	Cincinnati, OH, USA
IL-1β	35 kDa	AF5103	1:1000	--	Affinity	Cincinnati, OH, USA
TNF-α	25 kDa	AF7014	1:500	--	Affinity	Cincinnati, OH, USA
IL-10	30 kDa	DF6894	1:1000	--	Affinity	Cincinnati, OH, USA
goat anti-rabbit	--	5220-0336	1:5000	1:400	SeraCare	Massachusetts, USA

Table S3. Details of commercially available test kits.

Cytokines	Catalog number	Company	Area
TC	A111-1-1	Nanjing Jiancheng Biotechnology Research Institute	Jiangsu, China
TG	A110-1-1	Nanjing Jiancheng Biotechnology Research Institute	Jiangsu, China
LDL-C	A113-1-1	Nanjing Jiancheng Biotechnology Research Institute	Jiangsu, China
HDL-C	A112-1-1	Nanjing Jiancheng Biotechnology Research Institute	Jiangsu, China

Table S4. Details of primer 341F-806R

Amplified Region/Primers Name	Forward primer sequences	Reverse primer sequences
V3-V4 region / 341F-806R	CCTAYGGGRBGCASCAG	GGACTACNNGGGTATCTAAT

Table S5. Relative abundance (log10) of top 10 phylum among NCD, HFD, and HFD+TF groups.

Taxa	NCD	HFD	HFD+TF
p__Firmicutes	0.522357494	0.499754852	0.475164626
p__Bacteroidota	0.354110565	0.078315343	0.123943789
p__Verrucomicrobiota	0.006373847	0.213014716	0.207395171
p__Actinobacteriota	0.010205698	0.132481727	0.037048268
p__Proteobacteria	0.030677438	0.040626674	0.098173836
p__Desulfobacterota	0.032951657	0.016100563	0.036455387
p__Campilobacterota	0.014886138	0.004623868	0.0158177
p__Patescibacteria	0.024107473	0.001829181	0.000158403
Unassigned|p__uncultured	0.000358293	0.005868465	4.53×10^{-5}
p__Deferribacterota	0.002930461	0.000196118	0.003616121

Data are presented as the mean.

Table S6. Relative abundance (log10) of the top 30 genus among NCD, HFD, and HFD+TF groups.

Taxa	NCD	HFD	HFD+TF
Bifidobacterium	0.000377	0.013336	0.00836822
Roseburia	0.006517	0.013604	0.005756828
Faecalibaculum	0.000155	0.022908	0.004942183
f__Atopobiaceae | g_*uncultured*	0.003432	0.031715	0.01175805
[Ruminococcus]_torques_group	0	0.037609	0.025797108
Lactobacillus	0.012137	0.035441	0.021574529
Coriobacteriaceae_UCG-002	0.000173	0.061615	0.01284877
Dubosiella	0.018778	0.124056	0.037125206
Alistipes	0.009497	0.002772	0.005720622
Helicobacter	0.014886	0.004564	0.0158177
Alloprevotella	0.015018	0.009663	0.014387545
f__Desulfovibrionaceae | g_*uncultured*	0.024213	0.015244	0.033051979
f__Lachnospiraceae | g_*uncultured*	0.104369	0.059635	0.084415379
[Eubacterium]_xylanophilum_group	0.024149	0	0
Candidatus_Saccharimonas	0.024107	0.001754	0.000158403
Parasutterella	0.019137	0.005721	0.008255075
Clostridia_UCG-014	0.027257	0.008773	0.004000815
f__Oscillospiraceae | g_*uncultured*	0.031277	0.003741	0.009114978
f__Ruminococcaceae | g_*uncultured*	0.053714	0.001346	0.002172388
Lachnospiraceae_NK4A136_group	0.142476	0.002961	0.00170623
Muribaculaceae	0.268316	0.011514	0.011300944
Erysipelatoclostridium	0.000343	0.006736	0.011133489
Lachnoclostridium	0.001369	0.007901	0.00968523
[Clostridium]_innocuum_group	0	0.004481	0.031621823
Colidextribacter	0.012729	0.010745	0.015048313
Blautia	0.003262	0.025484	0.046398588
Escherichia-Shigella	0.000351	0.012793	0.077776018
[Eubacterium]_coprostanoligenes_group	0.006219	0.039514	0.070385373
Bacteroides	0.016764	0.0243	0.079676857
Akkermansia	0.006374	0.212566	0.207395171

Data are presented as the mean.

Table S7. The differential metabolites of serum among NCD, HFD, and HFD+TF groups.

**Group**	**NCD**	**HFD**	**HFD+TF**	**NCD *vs* HFD**		**HFD *vs* HFD+TF**	
				**VIP**	***P*-value**	**VIP**	***P*-value**
Diethyl phthalate	57016.8844	444375.348	39167.8645	1.679	0.001	3.727	0.004
Indolelactic acid	180693.288	622357.813	252641.206	1.683	0.007	3.337	0.027
N-palmitoyltaurine	95146.9121	349411.435	181219.889	1.39	0.000	2.169	0.022
Pseudouridine	633558.255	1776486.47	616402.034	2.327	0.036	5.920	0.012
Succinate	91796.3841	174989.017	82526.3074	1.075	0.027	1.653	0.015
5-aminovaleric acid betaine	8621185.47	21119442.4	13109624.2	2.576	0.000	1.404	0.025
Arachidonoylthiophosphorylcholine	28589053.9	52214191.1	31495357.1	4.268	0.013	4.407	0.037
D-pyroglutamic acid	2828539.98	6990248.42	3967753.47	1.851	0.017	1.858	0.017
L-palmitoylcarnitine	3082604.71	16036106.4	9825240.84	4.320	0.000	2.081	0.031

Data are presented as the mean. Differences were considered statistically significant at $p < 0.05$ and VIP > 1.
